# Abstract of Meteorological Observations for September, 1854, Made at Philadelphia, Pa.

**Published:** 1854-11

**Authors:** James A. Kirkpatrick


*Abstract of Meteorological Observations for September,* 1854, *made at Philadelphia, Pa. Latitude* 39° 57′ 28″ *N., Longitude* 75° 10′ 40″ *W. from Greenwich.* By PROF. JAMES A. KIRKPATRICK.

| 1854. Sept. | BAROMETER. Daily Mean | BAROMETER. Mean Daily Range. | THERMOM. Daily Mean | THERMOM. Mean Daily Range | Dew Point 2 P. M. | Rel. Humid. 2 P. M. | Rain. | Prevailing Winds. | General Remarks. |
|---|---|---|---|---|---|---|---|---|---|
| | Inches. | Inches. | Deg. | Deg. | Deg. | Hunds. | Inch. | Points. | |
| 1 | 30.069 | .031 | 78.0 | 4.7 | 65.0 | 0.65 | | NE. | Cloudy. |
| 2 | 30.014 | .055 | 73.3 | 5.3 | 66.3 | .78 | 0.008 | NE. | Cloudy; m. drizzling. |
| 3 | 29.940 | .074 | 81.3 | 8.0 | 68.2 | .67 | | SW. | Cloudy. |
| 4 | 29.952 | .036 | 81.3 | 3.3 | 57.7 | .52 | | NE. | M. and aft. clear; ev. cloudy. |
| 5 | 29.971 | .035 | 84.2 | 2.8 | 66.7 | .63 | | SW. | Aft. clear; m. and ev. clear. |
| 6 | 29.912 | .059 | 85.2 | 1.7 | 69.7 | .64 | | SW. | Cloudy. *Therm. highest* 94°. |
| 7 | 29.918 | .058 | 80.7 | 4.5 | 57.0 | .54 | | (Var.) | M. and ev. clear; aft. cloudy. |
| 8 | 29.969 | .082 | 80.5 | 4.2 | 52.0 | .45 | | (Var.) | Clear. |
| 9 | 29.752 | .218 | 85.0 | 4.5 | 63.0 | .57 | | SW. | Cloudy. |
| 10 | 29.722 | .110 | 61.7 | 23.3 | 57.7 | .94 | 4.452 | N. | Cloudy. M. heavy rain; high wind. *Bar. lowest* 29.657 in. |
| 11 | 29.986 | .264 | 61.5 | 7.8 | 53.0 | .69 | | (Var.) | M. clear; aft. and ev. cloudy. |
| 12 | 29.920 | .074 | 70.0 | 8.5 | 66.0 | .86 | 0.006 | SW. | M. and aft. cloudy; ev. clear. |
| 13 | 30.040 | .153 | 72.5 | 3.8 | 58.3 | .65 | | (Var.) | Cloudy. |
| 14 | 30.031 | .142 | 70.2 | 3.0 | 66.0 | .86 | 0.417 | (Var.) | Cloudy; aft. and ev. rain. |
| 15 | 29.914 | .164 | 72.0 | 5.2 | 55.7 | .57 | | NW. | M. and aft. cloudy; ev. clear. |
| 16 | 30.144 | .230 | 63.3 | 8.7 | 42.3 | .47 | | NW. | Clear. |
| 17 | 30.288 | .144 | 59.7 | 3.7 | 43.7 | .50 | | N. | Clear. |
| 18 | 30.197 | .092 | 65.5 | 5.8 | 49.7 | .54 | | (Var.) | Clear. |
| 19 | 29.866 | .330 | 73.0 | 7.5 | 60.8 | .66 | | (Var.) | Cloudy; ev. drizzling. |
| 20 | 29.878 | .157 | 65.5 | 8.5 | 67.7 | .48 | | NW. | M. cl'y; aft. & ev. cl'r; night *hoar frost* |
| 21 | 30.248 | .370 | 52.5 | 13.0 | 36.0 | .46 | | N. | M. and aft. cl'y; ev. cl'r. *Therm. low-* |
| 22 | 30.380 | .132 | 57.3 | 4.8 | 44.0 | .54 | | (Var.) | Clear [*est* 47°. |
| 23 | 30.287 | .094 | 60.0 | 2.7 | 48.0 | .56 | | SW.W. | M. and aft. cl'y; ev. cl'r. *Bar. highest* |
| 24 | 30.036 | .251 | 63.0 | 3.0 | 54.3 | .63 | | SW. | Clear. [30.388 in. |
| 25 | 29.945 | .091 | 69.5 | 6.5 | 57.3 | .65 | | SW. | M. and aft. cloudy, ev. clear. |
| 26 | 29.957 | .012 | 71.3 | 1.8 | 60.7 | .68 | | (Var.) | M. and aft. cloudy; ev. clear. |
| 27 | 29.928 | .033 | 74.8 | 3.5 | 63.3 | .67 | | E. | M. and aft. cloudy; ev. clear. |
| 28 | 29.914 | .045 | 74.7 | 2.8 | 57.7 | .58 | | (Var.) | M. cloudy; aft. and ev. clear. |
| 29 | 30.142 | .229 | 63.3 | 11.3 | 50.3 | .49 | | N. | M. and ev. clear; aft. cloudy. |
| 30 | 30.278 | .136 | 55.0 | 8.3 | 38.7 | .46 | | SW. | Clear. |
| Means for Sept. 1854 | 30.020 | .130 | 70.0 | 6.1 | 56.5 | .61 | 4.883 | W. 18—100. | |
| Means for Sept. 1853 | 29.976 | | 68.3 | | 54.2 | | 4.460 | | |
| Means for Sept. 1852 | 30.034 | | 65.9 | | 47.5 | | 1.290 | | |
| Means for Sept. 3yrs | 30.010 | | 68.1 | | 52.7 | | 3.544 | S. 86° W. 42—100. | |

The extreme range of the barometer during the month was 0.731 of an inch, and of the thermometer 47°.

